# Stability of Silver and Pyrex in Perchloric Acid-Silver Perchlorate Solutions and in Conductivity Water

**DOI:** 10.6028/jres.064A.011

**Published:** 1960-02-01

**Authors:** D. Norman Craig, Catherine A. Law, Walter J. Hamer

## Abstract

The stability of mint silver, purified mint silver, and Pyrex fritted crucibles in aqueous solutions of perchloric acid, in aqueous solutions of perchloric acid containing silver perchlorate, and in conductivity water at room temperature was determined. The stability of the silver in various states of subdivision was studied. The corrosion current-density for mint silver in sheet form is 1.1 × 10^−8^ amp cm^−2^ for 20 percent aqueous solutions of perchloric acid, 1.3×10^−9^ amp cm^−2^ for 20 percent aqueous solutions of perchloric acid containing 0.5 percent silver perchlorate, and within the limits of detection is zero for conductivity water. Pyrex has high stability at 25° C in 20 percent aqueous solutions of perchloric acid and in 20 percent aqueous solutions of perchloric acid containing 0.5 percent silver perchlorate. Finely-divided silver contained in Pyrex crucibles was found to be highly stable when the crucibles were filled repeatedly with aqueous solutions of perchloric acid containing silver perchlorate, rinsed with conductivity water, and dried at 105° C. These observations are important in the determination of the faraday by the anodic dissolution of silver in aqueous solutions of perchloric acid which is now underway at the National Bureau of Standards.

## 1. Introduction

As part of a program for the determination of the faraday at the National Bureau of Standards by anodic dissolution of metallic silver in aqueous solutions of perchloric acid it is essential to know the extent and rate of solution or the stability of silver at room temperature in: (1) Aqueous solutions of perchloric acid, (2) aqueous solutions of perchloric acid containing silver perchlorate, and (3) conductivity water. Obviously metallic silver should not dissolve or otherwise react with perchloric acid solutions at a significant rate if the anodic dissolution of silver in aqueous solutions of perchloric acid or of perchloric acid-silver perchlorate is to be used in accurate determinations of the faraday; otherwise the loss in weight of the silver cannot be correlated accurately with the quantity of electricity passed through the coulometer. Thermodynamic data [1, 2]^1^ show that silver does not undergo chemical reaction with perchloric acid; the free energy change for the postulated reaction is positive and, therefore not a spontaneous one. However, thermodynamically, silver may dissolve in perchloric acid solutions containing oxygen or go into solution as a result of galvanic corrosion arising from local anodic and cathodic areas on the silver surface or by “air-line” corrosion in such solutions. Therefore it is important to study the rates of these reactions in order to establish their effect on the accuracy of the faraday determinations by the proposed method.

During a coulometric run silver is anodically dissolved and silver perchlorate is formed. Therefore, the stability of silver in solutions of perchloric acid containing silver perchlorate must be determined. Furthermore, at the end of a coulometric run the silver anode which remains undissolved must be washed free of coulometric solution with conductivity water, dried, and weighed. Accordingly, the solubility or stability of silver in conductivity water must also be determined.

During the anodic dissolution of silver in perchloric acid it has been observed that a small amount of silver invariably falls from the anode to the bottom of the coulometer vessel (Pyrex beaker). This dislodged silver (or sediment), identified as silver by spectroscopic analysis, must be collected, washed free of coulometric solution with conductivity water, dried, and weighed in suitable crucibles. Pyrex fritted-crucibles are used for this purpose. Obviously, then, it is also necessary to determine the effect of perchloric acid, perchloric acid-silver perchlorate solutions, and conductivity water on the stability of the crucibles.

It is the purpose of this paper to present data on the stability of silver in various states of subdivision and of Pyrex in aqueous solutions of perchloric acid (20 wt %), in aqueous solutions of perchloric acid (20 wt %) containing silver perchlorate (0.5 wt %), and in conductivity water at room temperature. Changes in the combined mass of a Pyrex crucible and finely-divided silver during reheating at 105° C and during rewashing and redrying at 105° C are also presented.

## 2. Materials

Mint silver in rolled sheet form obtained from the United States Mint in Philadelphia, Pa., and purified mint silver in rod form were used in these experiments. The purified silver was prepared from mint silver by electrolytic transport in acidified silver-nitrate solution; the purified silver collected at the cathode was leached first in hydrofluoric acid, then in conductivity water. It was dried in a silver dish and fused in quartz tubes. The resulting silver rods were soaked in hydrofluoric acid to remove any surface layer of silica. Spectroscopic analysis showed the mint silver contained less than 10-ppm total metallic impurities and similar analysis showed the purified rods contained less than 1-ppm total metallic impurity.

The perchloric acid was reagent grade. Solutions of the desired concentration were made by dilution with conductivity water having a conductivity of 0.6 to 1.0×10^−6^ (ohm cm)^−1^ at room temperature. The conductivity water was produced in a conductivity still wherein one third of the condensate is collected; the feed was distilled water treated with alkaline permanganate. The silver perchlorate used in some of the solutions was prepared from reagent grade silver oxide and perchloric acid solution. The perchloric acid-silver perchlorate solutions were always filtered before using them.

The Pyrex crucibles used to collect the finely-divided silver as well as the Pyrex breakers used in the experiments were soaked in nitric acid solutions and thoroughly rinsed with conductivity water before using them. Both the inside and the outside of the glassware were cleaned. The crucibles had a capacity of 17 ml and an inner surface area of 28 cm^2^, exclusive of the glass frit the diameter of which was 1.8 cm.

## 3. Procedure

Unless otherwise noted the silver was weighed, immersed in the respective solutions for specified time intervals, removed, washed with conductivity water, dried at 105° C, and reweighed. Heating at 105° C was at times repeated. A plastic-paneled cabinet in a room free of fumes was used to protect the silver and the solutions from dust.

A semi-microbalance was used in the weighings involving unpurified mint silver. A micro balance was used in the weighings involving purified mint silver. The weighings were made by double transposition, with sensitivity measurements interspersed, in a temperature-controlled room maintained at 25 ± 1° C and with a relative humidity of always less than 50 percent. The balances were supported on an Alberene slab anchored to a wall of the laboratory. All weighings were corrected for buoyancy using the density of the weights, the prevailing density of air, 10.50 as the density of silver, and 2.245 as the density of Pyrex. The weights were calibrated by the Mass and Scale Section of the National Bureau of Standards and were used periodically to check the performance of the balances.

## 4. Results

### 4.1. Stability of Silver in Aqueous Solutions of 20 Percent Perchloric Acid

Sheets of mint silver, about 0.03 cm in thickness and 100 cm^2^ in surface area were used in these studies. The sheets were only 60 percent immersed in the solution with 40 percent above the solution in order to include air-line corrosion if it occurred (the silver is used in this fashion in the coulometer, i.e., part of the silver protrudes into the coulometer solution). Each time the silver was reimmersed in the solution that part previously exposed to air was immersed. Results of these studies are given in table 1.

**Figure 1:**
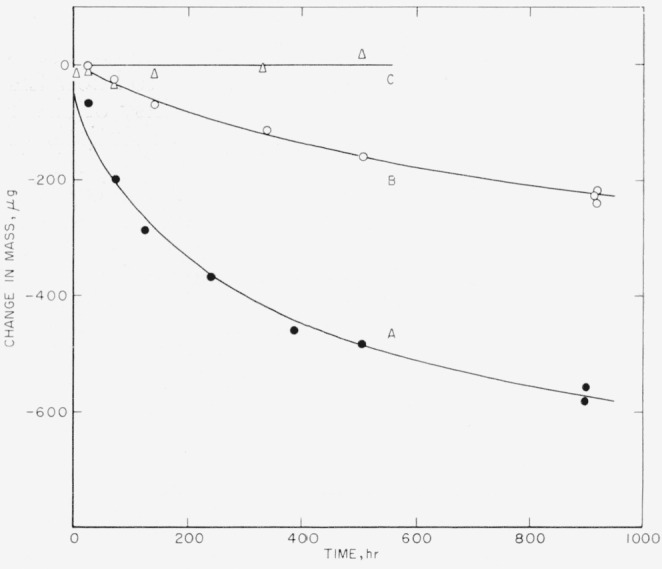
Stability of mint silver in sheet form at room temperature: in 20 wt percent of *HClO_4_ (A)*, in 20 wt percent *HClO_4_* containing 0.5 wt percent of *AgClO_4_ (B)*, and in conductivity water *(C)*.

**Figure 2:**
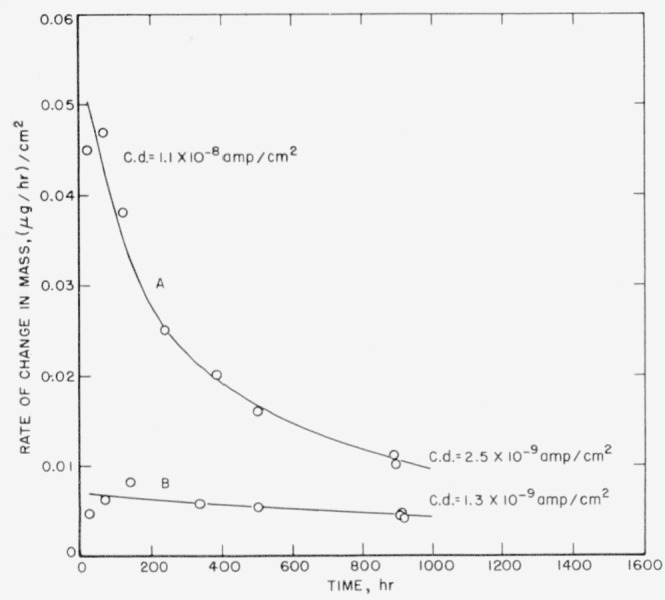
Rate of change in mass of mint silver in sheet form at room temperature: in 20 wt percent of *HClO_4_ (A)* and in 20 wt percent of *HClO_4_* containing 0.5 wt percent of *AgClO_4_ (B)*.

These data show that mint silver is quite stable in 20 percent aqueous solutions of perchloric acid and that the rate of corrosion does not proceed at a constant rate but decreases with time as the Ag^+^ content of the solution increases (see curve A of fig. 1). The initial corrosion current per cm^2^ of surface area for mint silver in 20 percent perchloric acid at 25° C is 1.1×10^−8^ amp and decreases with time to 2.5×10^−9^ amp, or lower (see later), as the Ag^+^ content of the solution increases. Thus, for 1 cm^2^ of surface area only 40 microcoulombs of electricity would be dissipated per hour for mint silver in 20 percent perchloric acid and only 9 microcoulombs or less per hour (see later) for 20 percent perchloric acid containing minute quantities of Ag^+^ ion (see curve A of fig. 2). Since faraday determinations have shown that silver is readily dissolved anodically at rates higher than 360 coulombs per cm^2^ of silver surface per hour, even the higher corrosion current viz, 40 microcoulombs per hour for a solution containing minute quantities of Ag^+^ introduces an uncertainty of about 1 part in 10^7^, or 0.1 ppm.

### 4.2. Stability of Silver in Aqueous Solutions of 20 Percent Perchloric Acid Containing 0.5 Percent Silver Perchlorate

Sheets of mint silver, 0.03 cm in thickness and 100 cm^2^ in surface area and rods (or anodes) of purified mint silver (estimated dimensional surface area ranged from 5 to 11.4 cm^2^) were used in these experiments. The sheets of mint silver were 60 percent immersed in the solutions, as above, whereas in most cases the rods were completely immersed. Results for mint silver are given in table 2. These data again show that the presence of silver ions in small amount curtails the rate of corrosion of silver in aqueous solutions of HClO_4_ (see curve B of fig. 1). The apparent increase in weight during the first 3½ hr, shown in table 2, is inconsistent with other observations. Neglecting this value and starting with the second it may be seen that the loss in weight per hour is nearly constant, although a slight decrease in rate is still evident. The average rate is 0.33 *μ*g/hr or (0.0054 *μ*g/hr)/cm^2^ of surface area (see curve B of fig. 2).

Results for purified mint silver in the form of rods are given in table 3. The average loss in (mass/hour) / cm^2^ for these samples, excluding the first value which is abnormally high, is 1.7 times that for the average of the values given in table 2 for mint silver in the form of sheets. It can be seen in table 3 that the changes in mass of the silver rods or anodes are nevertheless extremely small and that for the experiments involving long immersion of the samples the changes in (mass/hour)/cm^2^ is nearly the same as that for the sheet silver. The values of change, (mass/hour)/cm^2^, in table 3 calculated for short periods of immersion magnify any small uncertainty in the determination of the mass of the silver. Even the first and abnormally high value of (0.43 *μ*g/hour)/cm^2^ given in this table would cause an error of only 1 in 10^6^ for a faraday determination if a current density as low as 0.1 amp/cm^2^ were used, neglecting the fact that in a determination the Ag^+^ ion concentration would increase rapidly after electrolysis was started.

### 4.3. Stability of Silver in Conductivity Water

Sheets of mint silver about 0.03-cm thick and 100-cm^2^ in surface area and rods of purified mint silver (anodes no. 1 and no. 2) were used in these experiments. Again the sheets were 60 percent immersed in the water whereas the rods were completely immersed. The data of table 4 show that the mint silver in sheet form is practically inert in conductivity water at 25° C. The changes in weight are random; the average spread in values is only 10 *μ*g in 14 g, or less than 1 ppm. The standard deviation of the individual observations is 15.1 *μ*g; the standard deviation of the mean is 5.7 *μ*g. The data in table 5 for several samples of purified mint silver likewise show that silver is inert in conductivity water, and therefore the washing of the silver in conductivity water should cause no significant error in the determination of the faraday by the proposed method.

Figure 1 shows the stability of mint silver in sheet form, in perchloric acid (curve A), in perchloric acid-silver perchlorate (curve B), and in conductivity water (curve C). It is evident that mint silver exhibits high stability in conductivity water and in perchloric acid solutions, especially those containing silver perchlorate.

### 4.4. Stability of Finely-Divided Silver in Aqueous Solutions Containing 20 Percent Perchloric Acid and 0.5 Percent Silver Perchlorate

In section 4.2 it was shown that silver in sheet or rod form is highly stable in aqueous solutions containing 20 percent perchloric acid and 0.5 percent silver perchlorate. In this section data are given on the stability of finely-divided silver in a similar solution. The finely-divided silver used was that which is produced in a coulometer during a coulometric run, i.e., the sediment that falls from the anode during anodic dissolution; no attempt was made to measure the state of subdivision of the silver (see later). The finely-divided silver was collected in Pyrex fritted-crucibles of 17-ml capacity. The crucibles were then filled with 12 to 14 ml of solution, placed in the cabinet, and allowed to stand at room temperature for specified times. The solution was then drawn from the crucibles by suction and the crucibles dried at 105° C to constant weight. Since the changes in weight may have arisen not only from the solubility of the silver but also from the solubility of the crucible in the perchloric acid-silver perchlorate solutions the latter was also determined. As noted in the next section the crucibles lose weight at the rate of 0.5 *μ*g per hour in the perchloric acid-silver perchlorate solutions. Using this rate the results given in table 6 were obtained.

The data of table 6 show that, on a weight basis, finely divided silver is considerably more soluble or less stable than the bulk silver in sheet or rod form. This fact is to be expected since finely-divided silver, on a weight basis, has a much greater surface area than the bulk silver. Using the data obtained for the rate of solution of sheet silver, micrograms per hour (table 2) as a basis, the surface area of the sediment or finely-divided silver formed from sheet silver was calculated to be about twice that of the sheet silver and the sediment formed from purified silver rods from 100 to 900 times that of the sheet silver. Visual inspection also showed that sediment from sheet silver was much less finely-divided than sediment from the purified rods. However, in the faraday determinations that have been made it has been found that the weight of the sediment from the corrosion of a given weight of the purified rods or anodes is about 1/20 that of the sediment from sheet silver.

In the faraday determinations filtering of the sediment is finished within 2 hours after the end of electrolysis. The data in table 6 show that the maximum loss in weight of the sediment from purified anodes is 1.38 ug/hr. This would cause an error no greater than 2.5 ppm for a 5-hr run at 0.2 amp. The data presented thus far for finely-divided silver relates to its solubility or stability after is has been collected in a Pyrex crucible at the end of a number of faraday determinations. However, the question arises whether some of the sediment as it is formed at the anode during a faraday determination exists in an even finer state of subdivision than the sediment collected in the crucible and accordingly may have not only a greater rate of solubility than that collected in the crucible but may be also collodial and some of it transported from the anode chamber by electrophoresis. The latter question was investigated after a number of faraday determinations by filtering the solution in the beaker adjacent to and connected with the anode compartment by a syphon. In no case was a significant change in weight of the crucible found showing that migration of silver particles from the anode compartment does not occur, even though the rate of filtering was slower for successive runs owing to the closing of the pores of the frit by the accumulation of finely-divided silver.

In the process of corroding silver anodically for a faraday determination there is the possibility that some silver is formed in an extremely finely-divided and very reactive state which, because of its increased solubility, may not be collected in the crucible at the end of the run. Evidence that this does not occur and therefore does not introduce a significant error consists in the agreement between the values of the faraday obtained for runs in which the amount of the finely-divided silver collected varied widely and for runs in which the current density was also varied widely. Examination of the results obtained in the runs made, shows no evidence that this is a source of significant error in the experimental procedure.

### 4.5. Stability of a Pyrex Crucible Containing Finely-Divided Purified Mint Silver and Conductivity Water

After filtering the sediment at the end of faraday determinations it is of course necessary to wash the crucible containing the collected silver. Although this requires only a relatively short time, nevertheless tests were made to determine the effect of conductivity water on the mass of the crucible containing finely-divided silver that fell from purified anodes during several faraday determinations. In these tests the crucible containing the finely-divided silver was about two-thirds filled with conductivity water and allowed to stand for varying periods. The water was then withdrawn by suction and the crucible and silver washed with an additional 100 ml of conductivity water, dried at 105° C and weighed. The results of these tests are given in table 7. Since the weight of the crucible was very nearly that of the tare the weights in columns 2 and 3 are very nearly those of the finely-divided silver. These results show that the mass of the crucible and the contained silver is not changed by conductivity water even over prolonged periods of time.

### 4.6. Stability of a Pyrex Crucible in Aqueous Solutions of Perchloric Acid

A Pyrex crucible of known weight was two-thirds filled with a 20 percent perchloric acid solution or a 20 percent perchloric acid solution containing 0.5 percent silver perchlorate, allowed to stand at room temperature for a week or more in the cabinet, the solution was then withdrawn by suction and the crucible washed with 100 to 150 ml of conductivity water, and dried at 105° C, and reweighed. The pertinent data are given in table 8, and show that Pyrex crucibles are quite stable in perchloric acid and perchloric acid-silver perchlorate solutions at room temperature. For perchloric acid solutions the average loss in weight is 0.07 *μ*g/hr while for perchloric acid-silver perchlorate solutions the loss for a single observation is somewhat higher but probably not significantly so. The latter value, however, was used for the corrections made in table 6, mentioned above.

Since the filtering process at the end of a faraday determination does not require 2 hr even the largest value in table 8 for the loss in weight per hour, viz, 0.54 *μ*g, would not introduce an error for a 4-g run greater than 0.25 ppm in the value of the faraday. Wichers, Finn, and Clabaugh [3] have made comparative tests of the effects of various reagents on chemical glassware. In those tests they found that Pyrex is extremely resistant to attack by 60 percent perchloric acid solutions at the boiling point and somewhat less resistant to attack by distilled water at 100° C. Even if the surface area of the frit in the crucible were known, other differences in the experimental conditions employed by the above authors from those employed in the present work preclude the possibility of making strict comparisons of their results with those given in table 8. Although we are primarily concerned with the stability of the fritted crucible under the conditions prevailing in the faraday determinations it is, nevertheless, significant that the above authors found Pyrex to be highly resistant to 60 percent perchloric acid solutions and also to distilled water.

### 4.7. Changes in Mass of a Pyrex Crucible Containing Finely-Divided Silver During Reheating at 105° C and During Rewashing and Redrying at 105° C

In the foregoing sections the stability of a Pyrex crucible and the stability of bulk and finely-divided silver in perchloric acid and perchloric acid-silver perchlorate solutions and in conductivity water was considered. In the experiments with finely-divided silver the silver was collected in Pyrex crucibles, washed free of perchloric acid or perchloric acid-silver perchlorate solutions with conductivity water and dried at 105° C. The question arises whether reheating or rewashing and redrying at 105° C cause any significant change in the combined mass of the crucible and the finely-divided silver. Therefore, in a series of faraday determinations the finely-divided silver that fell from the corroding anodes was transferred from the anode vessel and collected by filtration in a Pyrex crucible of known weight. The silver anode was then suspended in the anode beaker and rinsed repeatedly with separate portions of conductivity water. Each portion of water and any silver that fell from the anode during this washing procedure were transferred to the crucible. The anode was then suspended in an oven and dried at 105° C for 1 hr. The crucible was finally washed with an additional portion of water and dried for 2 hr at 105° C. The silver anode and the crucible were then reweighed separately and the above washing procedure was repeated using 100 ml of conductivity water after which the silver anode and the crucible were again dried and weighed.

The data for the crucible and the finely-divided silver obtained in these operations are given in table 9. The changes in mass are shown in the bottom portion of the table. The maximum change in mass is −15 *μ*g for reheating and +43 *μ*g for rewashing and redrying. For a faraday determination in which 4 g of silver are corroded these changes are 4 and 11 ppm, respectively. The average change is −5 *μ*g for reheating and +8 *μ*g for rewashing and reheating. These changes for a 4-g run are 1 and 2 ppm, respectively.

The data for the silver anodes used in the above operations are given in table 10. The maximum change in mass of an anode is −14 *μ*g for reheating and +14 *μ*g for rewashing and reweighing. For a faraday determination in which 4 g of silver are corroded these changes are 3 ppm. The average change is −3 *μ*g for reheating and −1 *μ*g for rewashing and reheating. These changes for a 4-g run are less than 1 ppm.

Finally, if the average change, −5 *μ*g? shown in table 9 for reheating the crucible is combined with the average change, −3 *μ*g, shown in table 10 for reheating the sheet-silver anodes the average change in the calculated value of the faraday for a 4-g run would be −2 ppm. The average change, +8 *μ*g, shown in table 9 for rewashing and redrying the crucible if combined with the average, −1 *μ*g, shown in table 10 for rewashing and redrying the anodes would cause a change of +2 ppm in the calculated value of the faraday.

## 5. Conclusions

The above results show that the analytical operations required for the determination of the faraday by the anodic dissolution of silver in perchloric acid can be performed accurately. In the absence of an electric current, silver in various states of subdivision is highly stable in aqueous solutions of 20 percent perchloric acid, especially in aqueous solutions of 20 percent perchloric acid containing 0.5 percent or more of silver perchlorate, and in conductivity water at 25° C. The Pyrex apparatus and particularly fritted Pyrex crucibles needed in the analytical procedures are likewise highly stable in the above solutions.

## Figures and Tables

**Table 1:** Stability of mint silver at 25° C in 20 percent aqueous solutions of perchloric acid

Time^a^	True mass in a vacuum	Total change in mass	Rate of change in mass	

*Hours*	*Grams*	*μg*	*μg/hour*	(*μg/hr*)/*cm*^2^
0	14.139731	……………………	……………………	……………………
25.5	14.139662	−69	2.7	0.045
72.0	14.139531	−200	2.8	.047
125.5	14.139444	−287	2.3	.038
241.5	14.139363	−368	1.5	.025
387.0	14.139268	−463	1.2	.020
504.0	14.139248	−483	0.96	.016
895.0	14.139151	−580	.65	.011
898.0	14.139172	−559	.62	.010

a Time of immersion of silver in the solution.

**Table 2:** Stability of mint silver at 25° C in aqueous solutions containing 20 percent perchloric acid and 0.5 percent silver perchlorate

Time^a^	True mass in a vacuum	Total change in mass	Rate of change in mass	

*Hours*	*Grams*	*μg*	*μg/hour*	(*μg/hr*)/*cm*^2^
0	13.848807	……………	……………	……………
3.5	13.848845	+38^b^	(b)	(b)
24.5	13.848838	−7	0.33	0.0055
71.0	13.848819	−26	.38	.0063
141.5	13.848776	−69	.50	.0083
339.0	13.848731	−114	.34	.0057
504.5	13.848685	−160	.32	.0053
912.5	13.848617	−228	.25	.0042
915.0	13.848611	−234	.26	.0043
917.0	13.848625	−220	.24	.0040

Average			0.33	0.0054

a Time of immersion of silver in the solution.

b See context.

**Table 3:** Stability of purified mint silver at 25° C in aqueous solutions containing 20 percent perchloric acid and 0.5 percent silver perchlorate

Time^a^	Initial mass in a vacuum	Final mass in a vacuum	Estimated surface area	Change in mass	Rate of change in mass	

Anode No. 1						
*Hours*	*Grams*	*Grams*	*cm*^2^	*μg*	*μg/hr*	(*μg/hr*)/*cm*^2^
3	13.657300	13.657289	8. 6	−11	−3.70	−0.43
46	9.542583	9.542576	6. 3	−7	−0.15	−.024
49	5.833381	5.833374	5.0	−7	−.14	−.028
65	5.833374	5.833363	5.0	−11	−.17	−.034
17	5.833373	5.833368	5.0	−5	−.29	−.058

Average					−0.19	−0.036^b^

Anode No. 2						
65	20.483577	20.483569	11.4	−8	−0.12	−0.010
94	16.377775	16.377773	9.3	−2	−.02	−.002
94	12.274162	12.274163	7.3	+1	+.01	+.001
330	8.174357	8.174357	6.6	0	.000	.000
787	8.174074	8.174044	6.6	−30	−.04	−.006

Average					−0.04	−0.004

Anode No. 4						
259	7.487765	7.487762	6.0	−3	−0. 01	−0.005
907	7.487762	7.487685	6.0	−77	−.08	−.013

Average					−0.04	−0.009

Anode No. 5						
13	20.069326	20.069335	11.2	+9	+0.69	+0.062
3,550	11.256877	11.256821	6.8	−56	−0.016	−0.002

Average					+0.33	……………

Average of all						−0.039
Average omitting the first						−0.009
Average of those above 200 hr						−0.0052

a Time of immersion of silver in the solution.

b Omit first value.

**Table 4:** Stability of mint silver in conductivity water at room temperature (immersed area 60 cm^2^)

Time^a^	True mass in a vacuum	Deviations from average mass	Rate of change in mass	

*Hours*	*Grams*	*μg*	*μg/hour*	(*μg/hr*)/*cm*^2^
0	14.139733	+9	……………	……………
4.0	14.139718	−6	−3.75	−0.062
24.0	14.139723	−1	−0.04	<−.001
73.0	14.139700	−24	−.45	−.008
141.5	14.139718	−6	−.11	−.002
332.0	14.139728	+4	−.02	<−.001
502.5	14.139749	+25	+.03	<+.001

Average ±10				
Average mass=14.139724 g				
Standard deviation of mean=5.7 *μ*g.				

a Time of immersion of silver in water.

**Table 5:** Stability of purified mint silver in conductivity water at room temperature

Time^a^	Initial mass in a vacuum	Final mass in a vacuum	Estimated surface area	Rate of change in mass	

Anode No. 1					
*Hours*	*Grams*	*Grams*	*cm*^2^	*μg/hr*	(*μg/hr*)*cm*^2^
18	9.542576	9.542565	6.3	−0.61	−0.097
22	5.833363	5.833374	5.0	+.50	+.100
75	5.833374	5.833373	5.0	−.01	−.002

Anode No. 2					
236	8.174357	8.174354	6.6	−0.01	−0.002
21	8.174343	8.174342	6.6	−.05	−.007
16	8.174342	8,174348	6.6	+.37	+.057
16	8.174077	8.174061	6.6	−1.00	−.151

a Time of immersion of silver in water.

**Table 6:** Stability of finely-divided silver at 25° C in aqueous solutions containing 20 percent perchloric acid and 0.5 percent silver perchlorate

Time^a^	Initial mass of silver, in a vacuum	Final mass of silver, in a vacuum	Change in mass (assumed to be silver)	Change in mass (corrected for solubility of crucible)	Rate of change in mass of silver	

Mint Silver						
*Hours*	*Grams*	*Grams*	*μg*	*μg*	*μg/hour*	(*μg/hr*)/*cm*^2^^b^
285	2.384495	2.384309	−186	−43	0.15	0.0087
325	2.165701	2.165476	−225	−62	.19	.012

Purified Mint Silver Sample No. 1						
19	0.038370	0.038335	−35	−25	1.32	4.77
40	.038335	.038301	−34	−14	0.45	1.62

Sample No. 2						
146	0.064493	0.064218	−275	−202	1.38	2.96
71	.064201	.064144	−57	−21	0.29	0.62

Sample No. 3						
60	0.068271	0.068200	−71	−41	0.68	1.38

Sample No. 4						
309	0.069862	0.069524	−338	−183	0.59	1.17

Sample No. 5						
13	0.070480	0.070456	−24	−17	1.31	2.57
16	.071283	.071260	−23	−15	0.94	1.82

Sample No. 6						
20	0.071937	0.071912	−25	−15	0.75	1.44

a Time solution was in crucible.

b Calculated by assuming area was a weight percent of area of sheet silver per gram (7.22 cm^2^ per gram).

**Table 7:** Stability of a Pyrex crucible containing finely-divided purified mint silver in conductivity water

Time^a^	Initial mass of crucible and silver in a vacuum	Final mass of crucible and silver in a vacuum	Change in mass (assumed to be silver)

Sample No. 1			
*Hours*	*Tare plus grams*	*Tare plus grams*	Micrograms
16	0.038301	0.038307	+6
16	.038307	.038329	+22

Sample No. 2			
45	0.064218	0.064201	−17

Sample No. 3			
60	0.068265	0.068271	+6

a Time water was in crucible.

**Table 8:** Stability of a Pyrex crucible in aqueous solutions of perchloric acid (20%) with or without silver perchlorate (0.5%)

Time^a^	Initial mass of crucible in a vacuum	Final mass of crucible in a vacuum	Change in mass per day	Change in mass per hour

Perchloric Acid-Silver Perchlorate				
*Days*	*Grams*	*Grams*	*μg/day*	*μg/hour*
7	11.329483	11.329393	−12.9	−0.54

Perchloric Acid				
15	11.329393	11.329438	+3.0	+0.13
25	11.329438	11.329344	−3.8	−.16
15	11.329344	11.329280	−4.3	−.18

a Time solution was in crucible.

**Table 9:** Stability of a Pyrex crucible containing finely-divided silver during reheating at 105° C and during rewashing and redrying at 105° C

Time^a^	Mass of crucible and silver in a vacuum (before run)	Mass of crucible and silver in a vacuum (after run and one heating)	Mass of crucible and silver in a vacuum (after reheat)	Mass of crucible and silver in a vacuum (after rewashing and redrying)

Masses				
*Hours*	*Grams*	*Grams*	*Grams*	*Grams*
0	13.314242	13.380023	13.380010	13.380029
48	13.380053	13.494553	13.494556	13.494542
432	13.494369	13.572640	13.572634	13.572619
48	13.572619	13.607940	13.607931	13.607932
120	13.608002	13.644927	13.644926	13.644925
72	13.644966	13.713368	13.713353	13.713379
456	13.713243	13. 765658	13.765661	13.765704

Changes in Mass				
*Hours*		*Grams*	*Micrograms*	*Micrograms*
0		0.065781	**−**13	+19
48		.114500	+3	**−**14
432		.078271	**−**6	**−**15
48		.035321	**−**9	+1
120		.036925	**−**1	**−**1
72		.068402	**−**15	+26
456		.052415	+3	+43

Average			**−**5	+8

a Time between coulometric runs.

**Table 10:** Stability of sheet silver anodes during reheating at 105° C and during rewashing and redrying at 105° C

Time^a^	Mass of silver in a vacuum (before run)	Mass of silver in a vacuum (after run and one heating)	Mass of silver in a vacuum (after reheat)	Mass of silver in a vacuum (after rewashing and redrying)

Masses				
*Hours*	*Grams*	*Grams*	*Grams*	*Grams*
0	13.848625	8.321713	8.321709	8.321711
48	8.321700	2.062902	2.062902	2. 062899
432	14.139172	9.282122	9.282125	9.282119
48	9.282119	5.150961	5.150955	5.150948
120	5.096498	2.498740	2.498738	2.498736
72	3.866473	1.237163	1.237162	1.237159
456	14.468994	10.318351	10.318337	10.318351

Changes in mass				
*Hours*		*Grams*	*Micrograms*	*Micrograms*
0		5.526912	−4	+2
48		6.258798	0	−3
432		4.857050	+3	−6
48		4.131158	−6	−7
120		2.597758	−2	−2
72		2.629310	−1	−3
456		4.150643	−14	+14

Average			−3	−1

a Time between coulometric runs.

## Footnotes

1 Figures in brackets indicate the literature references at the end of this paper.
